# Demographic Variation between Colour Patterns in a Temperate Protogynous Hermaphrodite, the Ballan Wrasse *Labrus bergylta*

**DOI:** 10.1371/journal.pone.0071591

**Published:** 2013-08-23

**Authors:** David Villegas-Ríos, Alexandre Alonso-Fernández, Mariña Fabeiro, Rafael Bañón, Fran Saborido-Rey

**Affiliations:** 1 Department of Ecology and Marine Resources, Institute of Marine Research (IIM-CSIC), Vigo, Pontevedra, Spain; 2 Servizo de Planificación, Dirección Xeral de Desenvolvemento Pesqueiro, Consellería do Mar e Medio Rural (Xunta de Galicia), Santiago de Compostela, A Coruña, Spain; 3 Red Sea Research Centre, King Abdullah University for Science and Technology (KAUST), Thuwal, Saudi Arabia; Aristotle University of Thessaloniki, Greece

## Abstract

Fish populations are often treated as homogeneous units in typical fishery management, thereby tacitly ignoring potential intraspecific variation which can lead to imprecise management rules. However, intraspecific variation in life-history traits is widespread and related to a variety of factors. We investigated the comparative age-based demography of the two main colour patterns of *Labrus bergylta* (plain and spotted, which coexist in sympatry), a commercially valuable resource in the NE Atlantic. Individuals were aged based on otolith readings after validating the annual periodicity of annuli deposition. The relationships between the otolith weight and fish age and between otolith length and fish length were strong but differed between colour patterns. The fit of the growth models to the age and length data resulted in divergent growth curves between colour morphotypes and between sexes. Males and spotted individuals attained larger mean asymptotic sizes (*L_inf_*) than females and plain individuals, respectively, but converged to them more slowly (smaller *k*). Estimates of mortality based on catch curves from two independent datasets provided a global total mortality (*Z*) of 0.35 yr^–1^, although *Z* was larger in plain and female individuals. Overall, the results of this research have direct implications for management of *L. bergylta* and, as a precautionary measure, we recommend considering both colour patterns as two different management units.

## Introduction

Age-based demographic studies of marine fishes are one of the foundations of population biology and provide essential information for ecosystem and fisheries management [1]. Aged-based information enables assessment of population stability and exploitability and allows for the evaluation of management initiatives [2]. Demographic parameters have been found to differ between populations of the same species geographically separated by tens to thousands of kilometres [3], [4], [5], highlighting the need to investigate local populations rather than apply models based on parameters from other regions for management purposes [6]. The rationale behind this is that fish demography is influenced by a number of variables including temperature, density of competitors, presence of predators, resource availability, fishing pressure and habitat quality [4], [7]. Demographic variation may also result from the evolution of alternative life-history strategies within and among populations, i.e. life-history variants, which may confer species increased resilience to environmental fluctuations [8], [9]. Furthermore, some species have developed into reproductively isolated sympatric morphotypes which can eventually promote population evolutionary divergence and ultimately, speciation [10]. Failing to consider this level of intraspecific variation in the demographic parameters can result in inadvertent undesirable outcomes and can have important implications for the sustainability and productivity of fisheries [9], [11].

**Figure 1:**
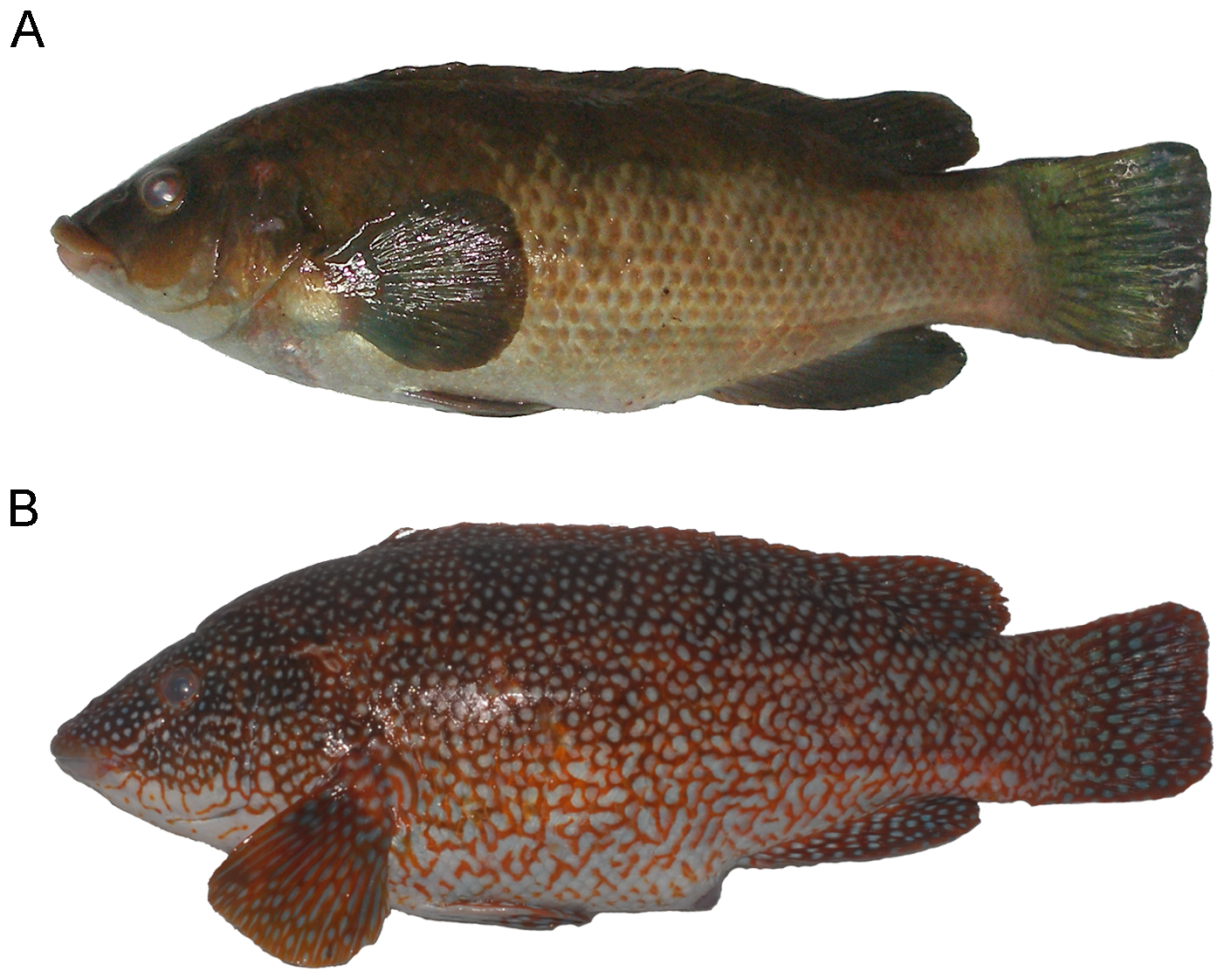
Colour patterns of *Labrus bergylta*. Example of (a) plain coloured individual (total length  = 39.2 cm) and (b) spotted coloured individual (total length  = 43.6 cm). Images are not in the same scale.

The ballan wrasse *Labrus bergylta* is a commercially valuable wrasse with a large contribution to both recreational and artisanal commercial catches throughout its distribution range. The species exists in coastal areas of the NE Atlantic and the Mediterranean, and is primarily associated with rocky reefs and kelp beds. The colour pattern of *L. bergylta* is highly variable [12], [13], but unlike other protogynous wrasses [14], [15], [16] it is not related to the sex of the individuals [13], [17], [18]. Two main colour patterns are usually differentiated and coexist in sympatry. Plain coloured individuals (Figure 1a) are characterized by a uniform, although variable, body colour (mainly greenish, brownish or reddish), darker in the back and whitish in the abdomen. Spotted coloured individuals (Figure 1b) display a dark orange or reddish body patterned with white dots. In NW Spain, there is a strong local belief that these are two different species and accordingly, spotted and plain individuals are usually commercialized separately (spotted individuals are noticeably more expensive). The presence of both colour patterns has been confirmed in Galicia [13], Azores Islands (J. Azevedo, pers. comm.), France (K. Nedreaas, pers. comm.), English Channel (C. Meyer, pers. comm.) and Norway (K. Nedreaas, pers. comm.). However, there are no reports on the biological significance of colour variation in *L. bergylta* in the scientific literature and the extent of ecological and/or life-history differences between colour morphs remains unknown.

*Labrus bergylta* has being described as a protogynous hermaphrodite species [13], [17]. However, management is based solely on a minimum landing size which varies along its distribution range (typically <23 cm), in spite of the evidence that in sequential hermaphrodite populations larger individuals should be also protected to ensure sex ratios are sufficient for effective mating and fertilization [19]. Management fails to consider the intraspecific variation in life-history traits. One reason for this mismatch between life-history and management is the existence of serious gaps in our knowledge of the age based-demographic parameters of *L. bergylta*, specially the potential differences between plain and spotted individuals. Previous studies were limited to report age based on scales and opercular bones, revealing that *L. bergylta* was a slow growing fish and males were larger at age than females. Maximum reported ages were 29 yr for males and 25 yr for females [20]. In this regard, the present study provides the first estimation of the otolith-based demographic parameters for the species. Our primary objective was to investigate the comparative demography of the plain and spotted individuals in the south of its distribution range. Since this is a protogynous fish [13], differences between sexes were also explored. As an essential first step, we validated the ageing criteria and the periodicity of annuli deposition in the otoliths. This information will be highly relevant to assess the status of the population and the fishing impacts, and to evaluate alternative management strategies of this important resource in the NE Atlantic.

## Materials and Methods

### Ethics statement

No specific permission for sampling was required for this study as all the individuals sampled were obtained from commercial fishing at the local fish markets. No protected species were sampled. Captive conditions and experiments with captive fish were carried out as part of an official research project, according to the European and Spanish legislation and specifically approved by Spanish authorities (09MMA022402PR).

Two different datasets were used to accomplish the objectives of this research. The main one (markets –MKT- dataset) was composed of information on 1583 individuals regularly obtained from the local fish markets in the south of Galicia, NW Spain (42°15’N, 8°50’W), from December 2009 to January 2012. These individuals were fished with gillnets at depths from 0 to 60 m. The sampling was conducted randomly to obtain a representative sample of the population, with the exception of the smaller size classes (typically under 15 cm) which are not captured by gillnets. For all the individuals in the MKT dataset, the colour pattern (plain vs. spotted), total length (TL;±1 mm) and total weight (TW; ±0.1 g) were recorded. Sex of the individuals was determined microscopically after histological processing of the gonads (see details in [13]) and sagittal otoliths were removed, washed in freshwater and stored dry until processing. The second dataset (UTPB dataset), composed by the historical (2000–2011) artisanal fishing monitoring data from the Coastal Fisheries Technical Unit of the Galician Regional Government (Xunta de Galicia), was used for mortality estimates. This dataset is considered a random sampling of the commercial catch. It included 10,163 records of *Labrus bergylta* (with no reference to the colour pattern or sex) measured (TL) to the nearest 1 cm. UTPB data were obtained by fisheries observers on board of randomly selected artisanal boats who registered all the individuals of *L. bergylta* captured in each fishing trip.

Data processing and all analyses were conducted in R [21]. In all statistical analyses, significance level was set to α = 0.05, and selection of the optimal models was based on Akaike’s Information Criteria [22].

### Validation of ageing criteria

To be useful for ageing purposes, otoliths must 1) grow continuously throughout the life of the fish, 2) display visible growth increments, and 3) those increments must be formed on a regular and determinable timescale [23]. Continuous growth of otoliths was assessed by analysing the relationship between fish age and otolith weight and between fish TL and otolith length using linear models. Both analyses included fish colour pattern and sex (and their interaction) as factors in order to assess intraspecific variation in these relationships. A strong relationship was expected if otoliths accreted calcium carbonate throughout the life of the fish. For this purpose, a subsample of 498 otoliths chosen randomly for each pair was weighed (±0.1 mg). Another random subsample of 243 complete and intact otoliths was photographed with a digital camera attached to a stereomicroscope and measured in length (±0.01 mm) with image analysis software.

A visual inspection of the whole otoliths evaluated the existence of visible growth increments. The rate at which those increments were formed in the otoliths was assessed using oxytetracycline (OTC) marking [24]. Eight individuals were injected with 50 mg of OTC per kg of fish on February 1^st^ 2011, and kept in 700 L tanks for a growth period of 433 d after OTC injection. They were maintained under controlled natural photoperiod and water temperature and fed *ad libitum* with mussels and sandworms 3 times per week. After the growth period, the 8 individuals were euthanized and otoliths removed and examined with a fluorescence stereomicroscope. Photos were taken with a digital camera attached to the microscope, and measurements (±0.01 mm) were made with image analysis software. The model of Cappo et al. [25] modified by Pears et al. [26] was used to obtain estimates of the periodicity of annuli formation. This model proposes that if each annulus (constituted by one opaque and one translucent zone) is formed in 1 year, then the distance from the OTC mark to the outer edge of the otolith, divided by the width of the last complete cycle, should be equal to the time elapsed from mark to death. In this model the periodicity of increment formation, P (d^–1^), is:
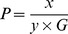



where x is the distance from the OTC mark to the edge of the otolith (mm), *y* is the distance of the last complete increment (mm) and *G* is the growth period (d).

Complementarily, marginal increment analysis and edge-type analysis [24] were undertaken. We measured and analysed the marginal increments for a subsample of individuals of age classes 5 (n = 68) and 6 (n = 68). For edge type analysis otoliths with indeterminate edge type (due to their inherent variability) were excluded. Thus, only otoliths classified as “G” (Table 1; [27]) from ages 3 to 12 were retained for analysis. After validating the annual nature of increment deposition, otoliths of 919 individuals from the MKT dataset covering the full range of fish TL, colour patterns, sexes and months were examined for age determination.

**Table 1:** Reader confidence index for age estimates of *Labrus bergylta* and number of otoliths assigned to each class.

Code	Qualitative meaning (pattern clarity)	Quantitative meaning (repeatability)	n
G	Pattern is very clear with no interpretation problems	Reader would always get the same age	252
FG	Pattern is clear with a few easy interpretation problems	Reader would get the same age most of the time	565
F	Pattern is fairly clear with some areas presenting easy and moderate interpretation problems	Reader would be within 1 yr all time for fish aged <10 and 2–3 years for fish >10	77
FP	Pattern is fairly unclear presenting a number of difficult interpretation problems	Reader would be within 2 yr all time for fish aged <10 and 3–4 years for fish >10	16
P	Pattern is very unclear presenting significant interpretation problems	Reader has little confidence in repeatability of age within 5 or more yr	9

### Age determination

Age readings were conducted on a stereomicroscope using reflected light over the whole otolith immersed in mineral oil. Age counting was made along a consistent axis near the sulcus. Age assignment was based on the annulus count, the edge type in relation to collection date and assigned birth date (by convention: January 1^st^). The primary reader examined each otolith (n = 919) on two occasions without prior knowledge of the fish characteristics, and assigned a reader confidence index (adapted from [27]) to the readings (Table 1). A second reader examined a random subsample of the otoliths (n = 222) on one occasion. The counts from both readers were compared, and the precision of age estimates calculated using the Index Average Percent Error (IAPE [28]). In addition, all the otoliths classified as “F”, “FP” or “P” were analysed two more times by the primary reader until consensus was reached. In the case that no agreement was achieved after these readings, these otoliths were considered unreadable and disregarded for the analysis.

### Demography

The relationship between fish TL and fish TW was described by a power equation. The relationship was linearized by log transforming data and a linear model was adjusted. Differences in the relationship between sexes and colour patterns were assessed by including these variables (and their interaction) as factors in the model.

Growth parameters were estimated using the iterative non-linear least squares method within the *nlstools* package using the Gauss-Newton algorithm [29] to fit the von Bertalanffy growth function (VBGF):
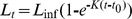
where *L_t_*  = size at age *t*; *L_inf_*  =  asymptotic length; *K* =  curvature parameter that represents the rate at which the asymptotic length is approached; *t* =  age of fish and *t_0_*  =  the age at which fish have theoretical length of 0 cm. Because the VBGF parameters estimate can be sensitive to the range of ages and sizes used and no fish of age 1 were collected, the intercept was constrained to 0 cm [30], [31]. Parameters *L_inf_* and *K* and their confidence intervals were obtained by bootstrapping with 1000 iterations [30]. Potential differences in growth curves between colour patterns, between sexes, and between sexes within each colour pattern were investigated using bivariate 95% confidence ellipses surrounding the *K* and *L_inf_* estimates [32]. Non-overlapping confidence regions indicate differences in the growth parameters [3], [33], [34]. For comparison, we fitted and calculated the parameters of the unconstrained VBGF, which is provided as Supporting Information. In addition, since the biological interpretation of the VBGF parameters has been questioned when comparing them between populations [35], [36], [37], the VBGF was reparametrized after Francis [38] (Text S1).

For the remaining demographic analysis, age was assigned to the 682 individuals in the MKT dataset that were not directly aged using sex- and colour-specific age-length keys created with the aged individuals. Differences in the mean size and age between sexes and colour patterns of the individuals of the MKT dataset were then compared using t-tests. Mean maximum age (longevity) and mean maximum size were calculated as the average age (yr) and average TL (cm) of the oldest 10% individuals [26] of the MKT dataset. Wilcoxon test was used to detect differences in longevity and mean maximum size between plain and spotted individuals.

Age-based catch curves were used to estimate the instantaneous rate of total mortality (*Z*) for *L. bergylta* [39], [40] under the following assumptions: (1) no trend in recruitment over time, (2) no trend in the fishing mortality rate over time, (3) constant natural mortality at age for the analysed ages, and (4) constant selectivity at age for the analysed ages [41], [42]. Catch curves were generated by plotting the logarithm of the number of individuals by age class. A linear model was then fitted to the descending limb of the catch curve. The slope of this regression is an estimate of *Z.* Linear models were fitted from a minimum age of one year older than the maximum catch frequency (assumed full selectivity) up to the oldest age class that was preceded by no more than 2 consecutive zero frequencies [31], [43]. The annual mortality rate (*A*), i.e., the proportion of the population that suffers mortality in a given year, was calculated as:
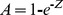



Catch curves were analysed with the *FSA* package [44].

A global value of *Z* for the species was calculated using data from 2011, including the dataset (MKT and UTPB) as a factor to test for differences between them (age of the individuals of the UTPB dataset was assigned from an age-key length created with the aged individuals of the MKT dataset). In addition, Z was estimated by cohort using the UTPB dataset. Eight cohorts (1997–2004) were selected based on the existence of a sufficient number of data and age classes. Sex- and colour-based specific mortality was investigated for the individuals in the MKT dataset. Due to the low number of males, an analysis of the interaction between sex and colour patterns was not possible. Therefore, we compared Z estimates by sex and colour pattern independently. Finally, colour-based differences in Z amongst females were evaluated.

## Results

### Validation of ageing criteria

**Figure 2:**
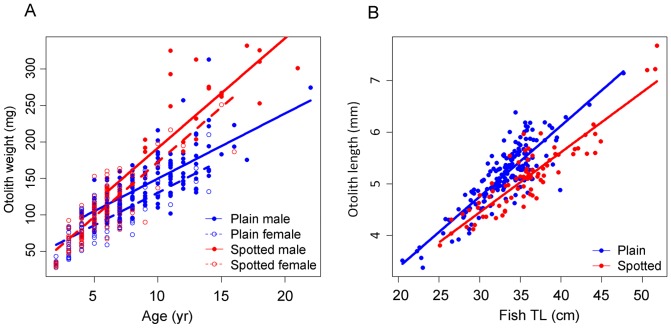
Relationship between otolith metrics and fish characteristics. Linear model fit on (a) otolith weight on fish age and (b) otolith length on fish total length (TL).

A significant positive linear relationship was observed between fish age and otolith weight (r^2^  = 0.79, F = 453.2, p<0.001, n = 498) but it differed significantly between plain and spotted individuals and between sexes (Figure 2a and Table 2). A positive significant relationship was also obtained between fish TL and otolith length (r^2^  = 0.75, F = 243.8, p<0.001, n = 243) with significant differences between colour patterns (Figure 2b and Table 2).

**Table 2:** Summary of the optimal linear models between fish metrics and otolith metrics. Plain individuals and males were considered the reference levels.

Dependent variable	Explanatory variables	Estimate	Std. Error	t value	p
Otolith weight	Intercept	60.414	4.324	13.971	<0.001
	Fish age	8.931	0.447	19.991	<0.001
	Colour pattern*	–19.119	5.092	–3.754	<0.001
	Sex**	–19.329	2.690	–7.187	<0.001
	Fish age x Colour pattern	6.111	0.646	9.456	<0.001
Otolith length	Intercept	0.641	0.220	2.915	0.004
	Fish TL	0.137	0.007	20.801	<0.001
	Colour pattern*	0.318	0.329	0.965	0.335
	Fish TL x Colour pattern	–0.021	0.009	–2.199	0.010

**Figure 3:**
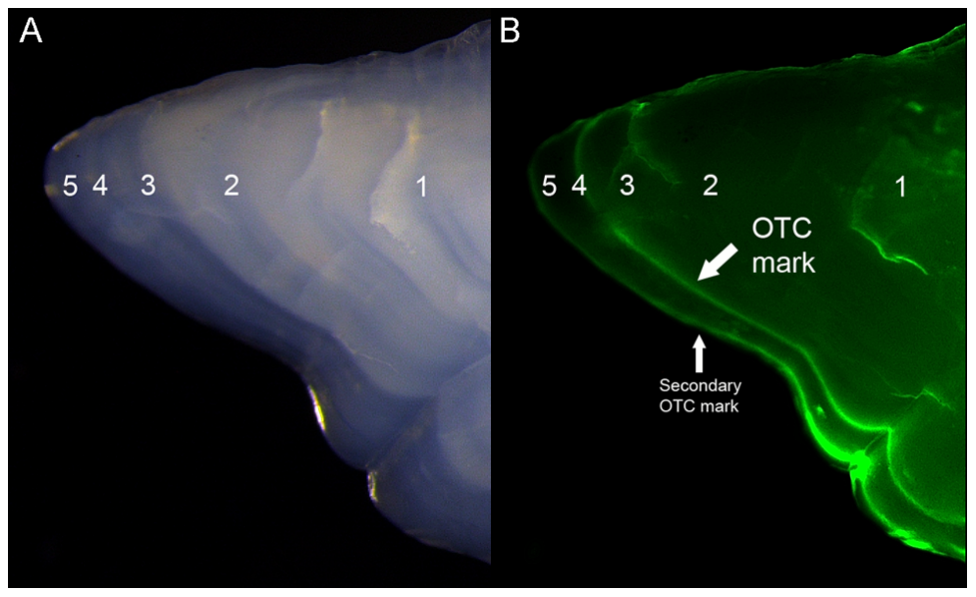
Validation of ageing criteria (I). Details of OTC-marked otolith showing (a) 5 annual bands in a captive individual and (b) OTC mark on the same area under fluorescent light. Note the presence of a secondary OTC mark irrelevant for the purpose of this study (OTC injected later as part of another experiment).

**Figure 4:**
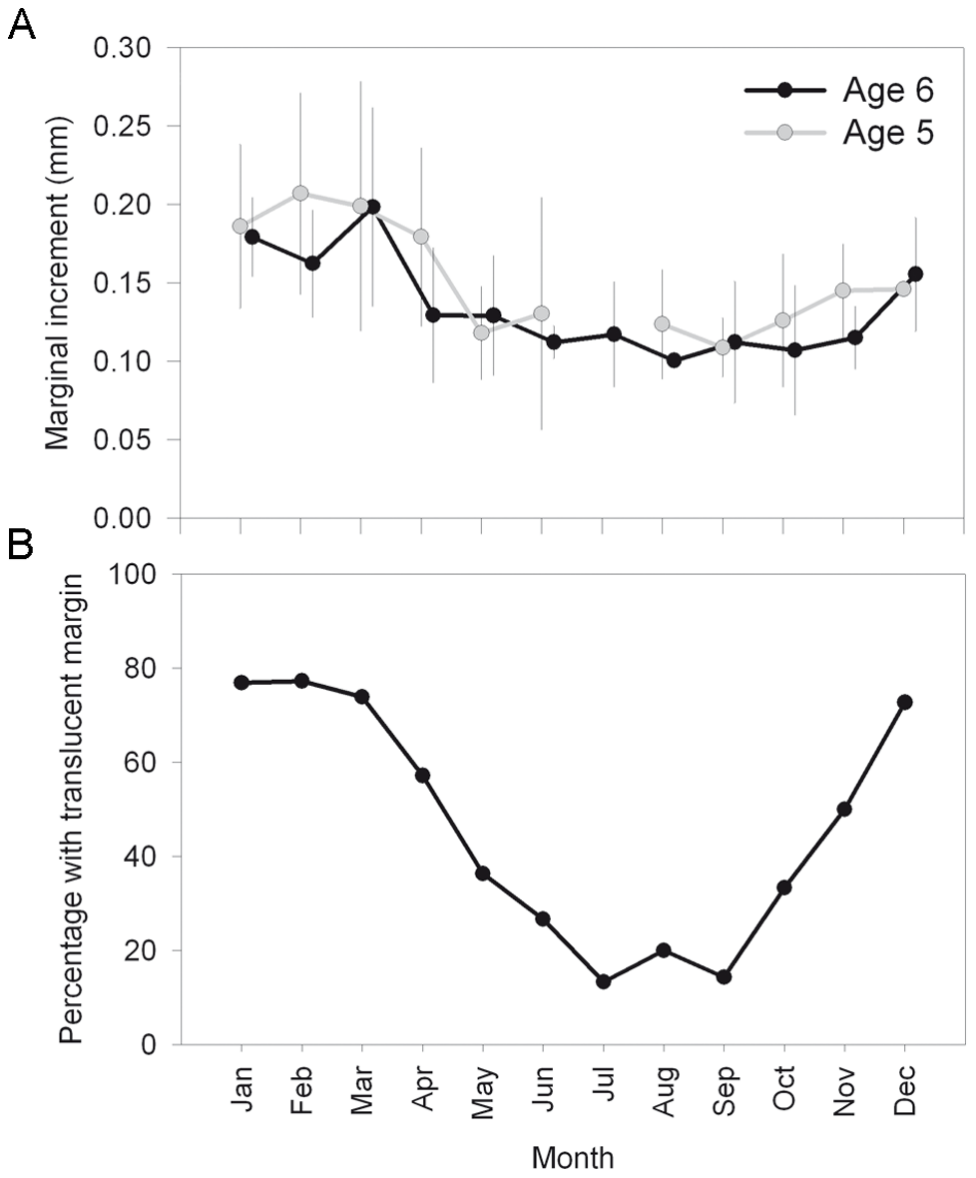
Validation of ageing criteria (II). Plot of (a) mean and standard deviation of marginal increment for the 5 and 6 yr classes and (b) monthly percentage of otoliths with translucent margin increments.

A visual inspection of whole otoliths under the stereomicroscope revealed a consistent and regular distribution of presumed annuli in the otoliths, with an alternation of opaque and translucent zones in all age classes. All the 8 injected individuals successfully revealed the OTC mark as a bright green line (Figure 3). Captivity conditions decreased the differentiation between opaque and translucent zones. Therefore, identification of annuli outside the OTC mark (and subsequent estimation of periodicity) was only possible for 6 individuals (Table 3). The calculated periodicity of annuli formation was 346±24 d (hereinafter all values expressed as mean ± standard deviation). The marginal increment displayed a cyclical trend (Figure 4a) with minimum values from June to September and a subsequent increase until the start of formation of a new opaque zone in March. The plot of monthly percentages of otoliths with translucent marginal increments (Figure 4b) revealed maximum values from December to March (slow growth season) and minimum values from July to September (rapid growth season).

**Table 3:** Details of the 6 individuals marked with OTC in the age validation experiment which successfully revealed the OTC mark.

Fish ID	Colour pattern	Sex	FishTL (cm) at start	Fish TL (cm) at end	Growth (cm)	Growth rate (cm y^-1^)	Location OTC mark	Zones outside OTC	Edge type at end	Age at end (yr)	Periodicity annuli formation (d)
OTC 1	Spotted	Female	33.5	36.1	2.6	2.2	T	1+	O	7	370
OTC 2	Spotted	Female	36.0	38.4	2.4	2.0	T	1+	O	5	309
OTC 3	Plain	Female	30.5	32.1	1.6	1.3	T	1+	O	6	371
OTC 4	Spotted	Female	31.0	33.4	2.4	2.0	T	1+	O	6	326
OTC 6	Plain	Female	26.0	28.7	2.7	2.3	O	1+	O	5	344
OTC 8	Plain	Female	23.5	27.1	3.6	3.0	T	1+	O	6	358

O =  opaque zone, T =  translucent zone.

### Demography

The log transformed fish TL and fish TW fitted to a positive linear model (r^2^  = 0.97, F = 16740, p<0.001, n = 1583), with significantly different results between sexes and colour patterns (Table 4).

**Table 4:** Summary of the optimal linear model between the log-transformed fish total weight (TW) and the log-transformed fish total length (TL).

Dependent variable	Explanatory variables	Estimate	Std. Error	t value	p
Log Fish TW	Intercept	–4.488	0.128	–34.923	<0.001
	log Fish TL	3.122	0.036	87.454	<0.001
	Sex*	–0.250	0.138	–1.797	0.073
	Colour**	–0.050	0.005	–9.408	<0.001
	log Fish TL x Sex	0.081	0.039	2.094	0.036

Plain individuals and males were considered the reference levels.

**Figure 5:**
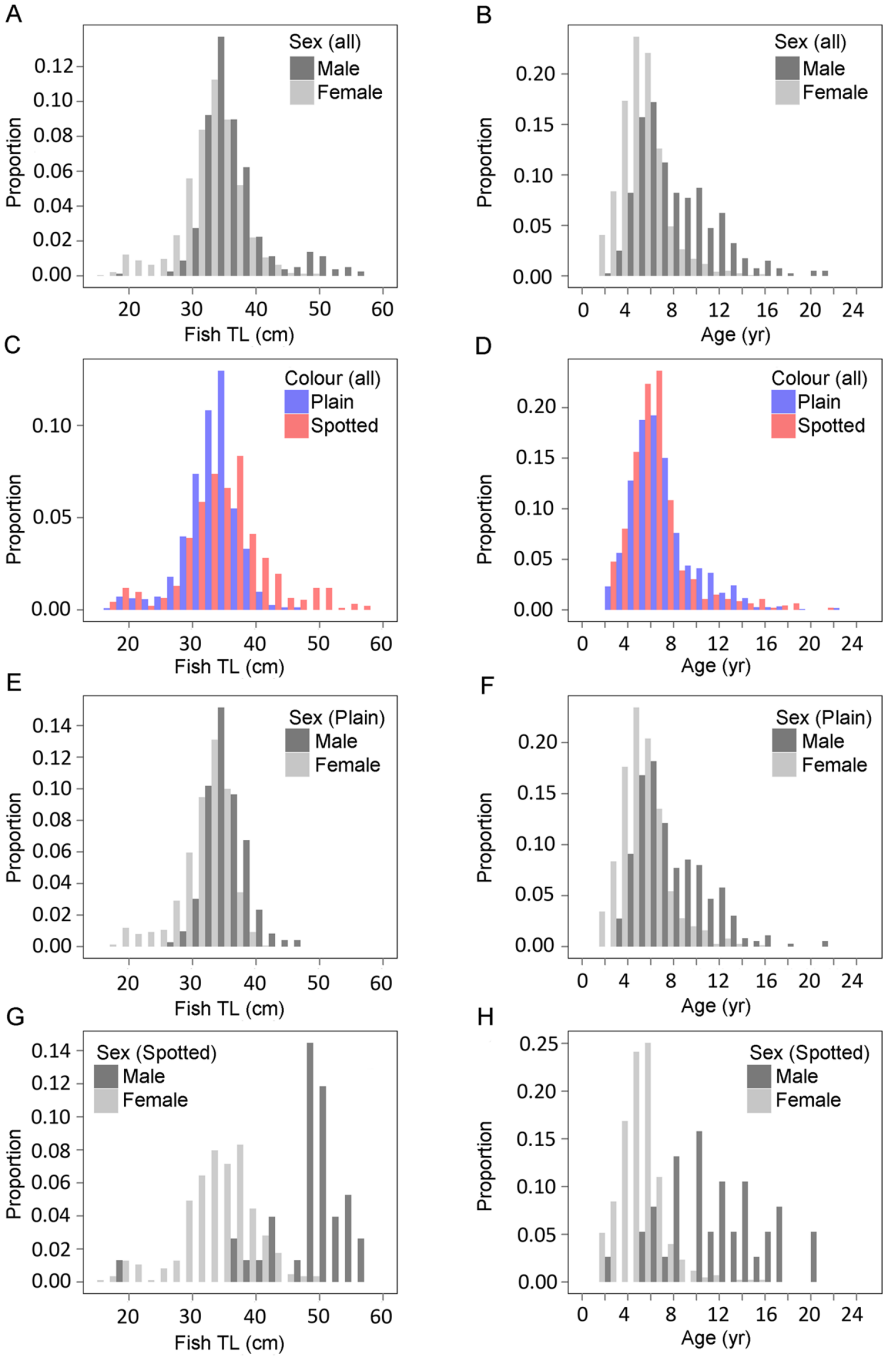
Size and age distributions of the sampled individuals. Independent plots are presented for the sex-specific size (a) and age (b) and colour-specific size (c) and age (d) distributions of all the individuals pooled. Additional plots are provided for sex-specific size (e) and age (f) distributions for plain individuals and sex-specific size (g) and age (h) distributions for spotted individuals.

Size and age distributions of the MKT samples showed different degrees of overlap between sexes and colour patterns (Figure 5). Males were larger (t = 14.91, p<0.001, df = 663.4) and older (t = 18.84, p<0.001, df = 498.5) than females. Yet females persisted in the larger and older groups. As for the colour pattern, spotted were larger than plain individuals (t = –5.74, p<0.001, df = 611.2). Conversely, plain individuals were older than spotted individuals (t = 4.45, p<0.001, df = 859.8). Differences in age and size structure by sex were additionally investigated separately for each colour pattern. Males were significantly larger (t = 18.32, p<0.001, df = 912.9) and older (t = 16.61, p<0.001, df = 424.2) than females within the plain group. These differences were even more noticeable in the spotted individuals, with males also significantly larger (t = 11.16, p<0.001, df = 33.4) and older (t = 9.17, p<0.001, df = 34.1) than females.

#### Age and growth

The precision of opaque-zone counts for *Labrus bergylta* was high with an IAPE of 1.8% (n = 222) between readers. There was good agreement among counts across all age classes, with 66.5% of the two counts being identical, 21.7% differing in one year and the other 11.8% differing in two to four years. The number of otoliths assigned within each class of the confidence index by the primary reader (Table 1) indicated that most otoliths were fair or good to read, with relatively minor and inconsistent differences between otoliths. Eighteen otoliths were considered as unreadable.

**Figure 6:**
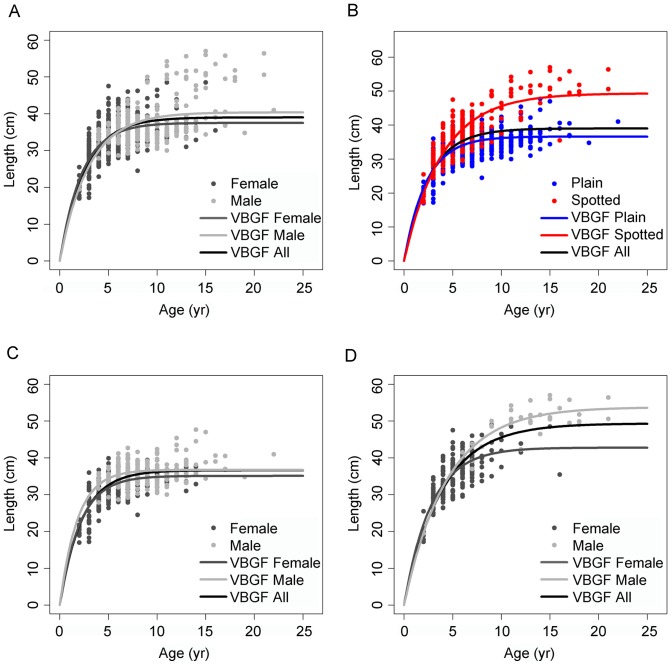
Growth curves of *Labrus bergylta*. Von Bertalanffy growth functions (VBGF) plotted by (a) sex, b) colour pattern, c) sex within plain individuals and d) sex within spotted individuals.

**Figure 7:**
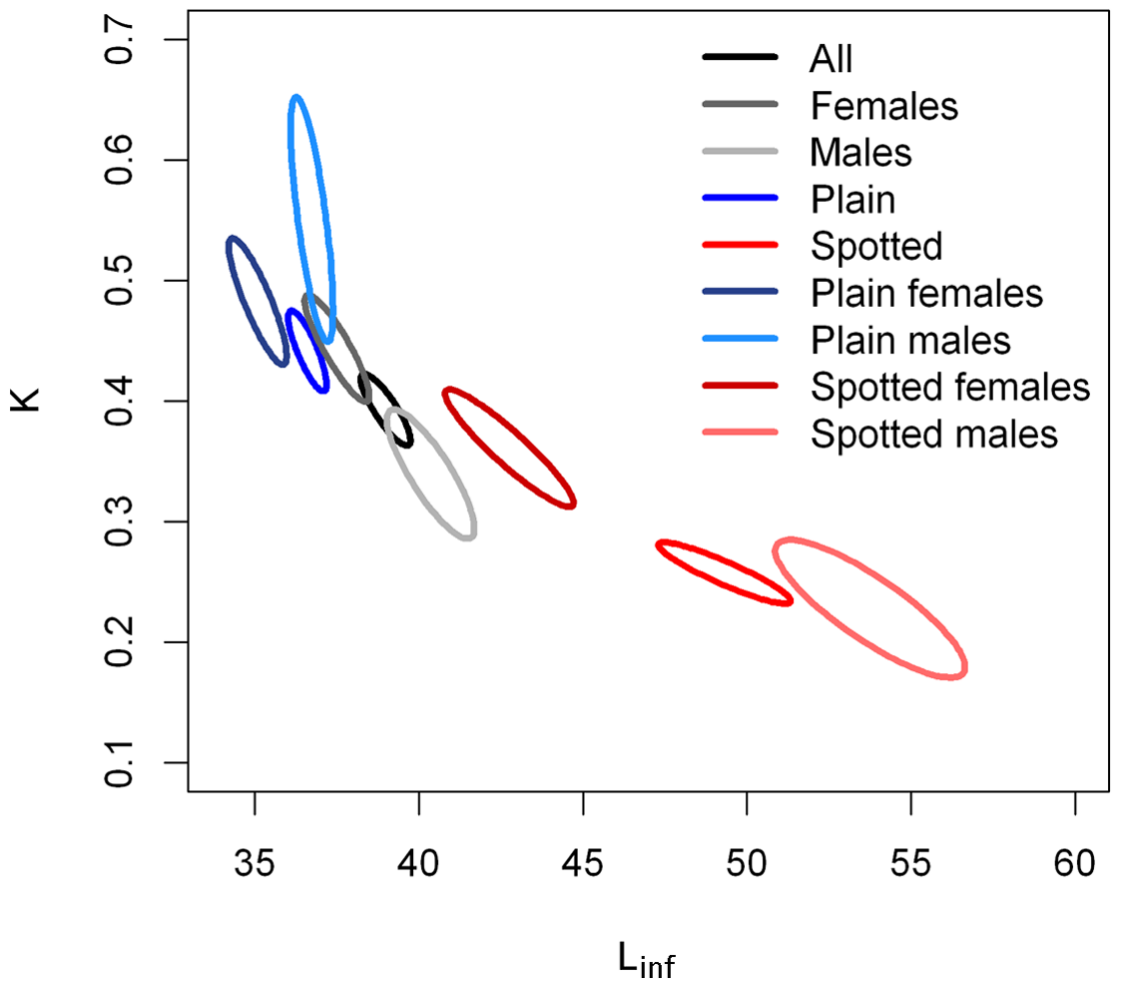
Bivariate 95% confidence ellipses around the von Bertalanffy parameters. Ellipses surround the growth coefficient (*K*) and mean asymptotic length (*L_inf_*) parameter estimates for the different sexes and colour patterns of *Labrus bergylta*. Non-overlapping confidence regions indicate differences in the growth parameters.

Size-at-age plots were created with the 901 individuals ultimately aged. Size variability increased with age in all the analysed groups (Figure 6). Fitting VBGF to each dataset gave the parameter values shown in Table 5, and the presence of non-overlapping 95% bivariate confidence intervals surrounding the parameter estimates (Figure 7) revealed significant differences between the compared datasets. Growth trajectories differed between sexes (Figure 6a) with males resulting in significantly larger *L_inf_* and smaller *K* estimations (Table 5, Figure 7). Partitioning the plot by colour pattern revealed increasingly divergent growth trajectories (Figure 6b) with plain individuals attaining significantly smaller *L_inf_* and larger *K* estimations than spotted individuals (Table 5, Figure 7). Within each colour pattern (Figures 6c and 6d), sex-specific differences were more evident in the spotted than in the plain individuals (Table 5, Figure 7). For comparative purposes, the parameters of the unconstrained VBGF are presented in Table S1, and the parameters of the rVBGF are presented in Table S2.

**Table 5:** Parameters of the constrained Von Bertalanffy growth functions with upper and lower 95% confidence intervals and Akaike Information Criteria (AIC) for each model.

Model	*L_inf_* - lower	*L_inf_*	*L_inf_* - upper	*K*- lower	*K*	*K*- upper	AIC
All	38.37	38.99	39.63	0.37	0.39	0.42	5195
Females	36.76	37.51	38.31	0.41	0.44	0.48	3225
Males	39.25	40.36	41.59	0.30	0.34	0.40	1937
Plain	36.15	36.60	37.08	0.42	0.44	0.47	3281
Spotted	47.67	49.32	51.08	0.24	0.26	0.28	1478
Plain females	34.43	35.09	35.81	0.44	0.48	0.53	1788
Plain males	36.23	36.74	37.29	0.48	0.55	0.66	1440
Spotted females	41.27	42.75	44.42	0.32	0.36	0.40	1238
Spotted males	51.68	53.76	56.24	0.19	0.23	0.28	180

#### Longevity and mortality

Estimated mean maximum age (longevity) for *L. bergylta* was 12.70 yr (±2.32) with no differences between colour patterns (W = 2940, p = 0.156, n = 158). Conversely, estimated mean maximum size was different for plain and spotted individuals (W = 554.5, p<0.001, n = 158), being 36.54±3.36 cm and 46.76±6.46 cm, respectively.

**Figure 8:**
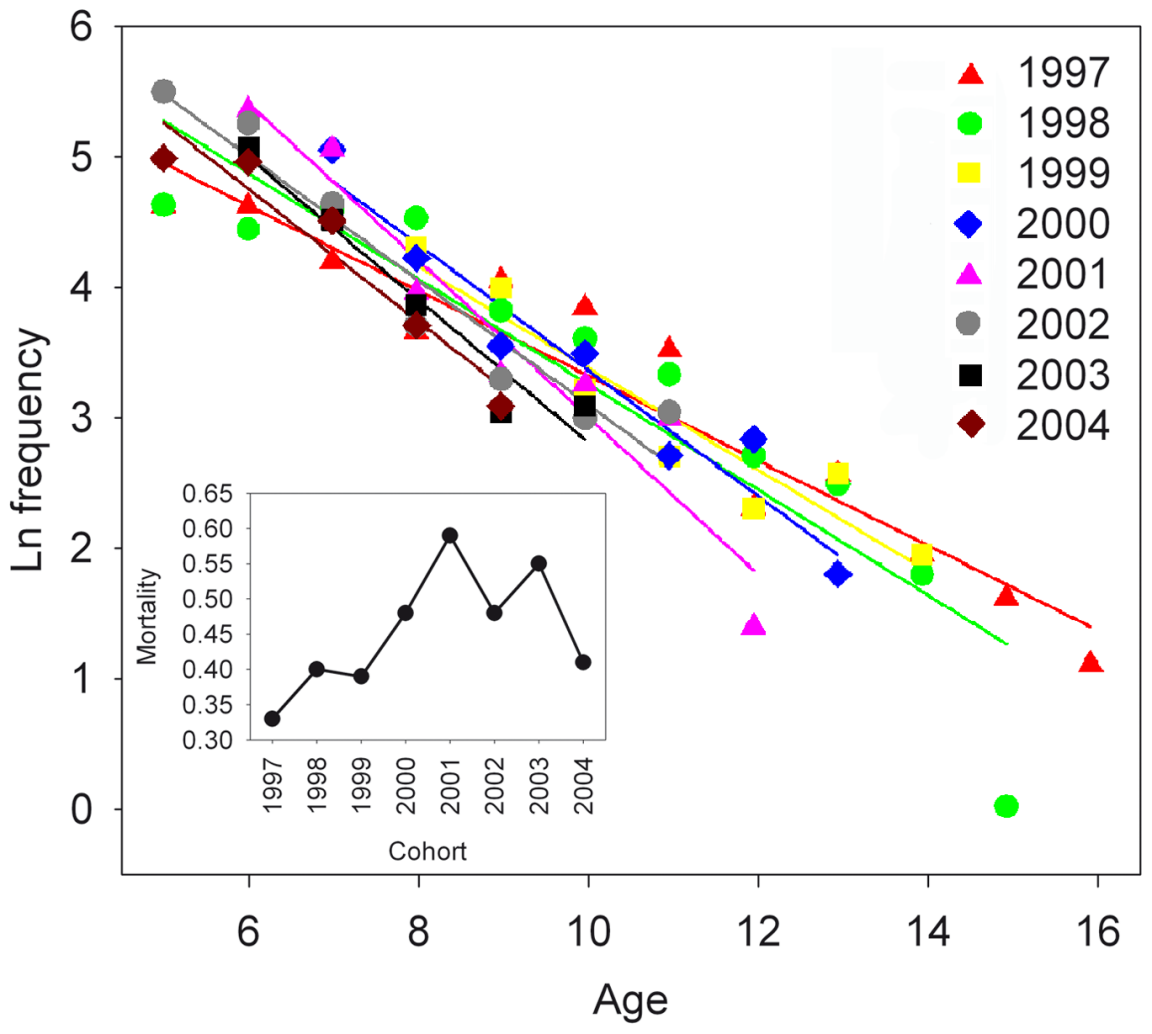
Mortality analysis of *Labrus bergylta*. Catch curves and estimates of mortality (inset) for the different cohorts analysed.

Estimate of Z with data from 2011 were 0.35±0.03 and with no significant differences between datasets (p = 0.666), and as a result A = 29.5%. Significant differences in mortality among cohorts were observed (r^2^  = 0.89, p = 0.029, F = 2.501, n = 61) (Figure 8). Analysis of the MKT dataset revealed significant differences in mortality between colour patterns (r^2^  = 0.90, F = 8.644, p = 0.008, n = 26) with Z_plain_  = 0.42±0.03 and Z_spotted_  = 0.28±0.05, and as a result A_plain_  = 34.3% and A_spotted_  = 24.4%. Differences in sex-specific mortality were also significant (r^2^  = 0.92, F = 16.07, p<0.001, n = 25), with Z_males_  = 0.32±0.05, Z_females_  = 0.51±0.04, A_males_  = 27.4% and A_females_  = 39.9%. Within the females, non-significant differences were found in Z estimates between colour patterns (p = 0.490).

## Discussion

This study represents the first estimate of the age-based demographic parameters of *Labrus bergylta* based on otoliths, and revealed striking demographic differences between the two main colour patterns of the species. Plain and spotted individuals displayed differences in the length-weight relationship, otolith and body growth, size and age structures and mortality. We estimated the age-based demographic parameters of *L. bergylta* using whole otoliths, which are preferred to scales and opercular bones as they are not reabsorbed or metabolically reworked and are not exposed to external damage [45]. Whole otoliths, together with the break and burn method, is one of the most rapid and widespread methods for fish age determination [46], [47].

The relationship between otolith weight and fish age and between otolith length and fish size showed that individuals accreted calcium carbonate in the otoliths throughout the lifetime of the fish. The robust relationship between fish size and otolith length permits the back calculation of fish body size at younger ages in individual growth studies [48], but specific parameters for each colour pattern should be used. The whole otoliths under reflected light showed a clear internal structure of alternating narrow translucent and wide opaque increments, as observed in other labroids [49]. The analysis of fish marked with OTC confirmed that *L. bergylta* produced 1 annulus per year on the otoliths. This result was confirmed by the presence of a single yearly minimum and maximum in the edge type and marginal increment analysis. The maximum percentage of fish with translucent margins found in winter (slow growth period) corresponds to the coldest water temperatures in the study area [50].

The observed maximum age in Galicia in this study (22 yr) is smaller than previously reported in the Irish Sea (29 yr; [20]) and represents a modest longevity within the family Labridae [30], [51]. Reasons behind this difference in longevity may be related to the lower water temperature in the Irish Sea (http://iridl.ldeo.columbia.edu last accessed: 23/05/2013). It’s recognized that adult longevity increases when there is a decline in average water temperature during the individuals’ development [52], [53]. Variation could also be related to differing selective pressures between populations from each location [3], different hard structures used for ageing (otoliths vs. opercular bones) or sampling bias.

Size-at-age plots revealed that *L. bergylta* invested a relatively small proportion of its life span in an initial rapid somatic growth as other labrid species [4], [49], [54]. Growth curves revealed sex-specific differences with males attaining larger sizes (*L_inf_*) than females but at a slower rate (*K*), as observed in other labroids [30], [54]. This might be explained by a growth spurt after sex change, as previously suggested [20], [55]. The fit of VBGF separately for each colour pattern also revealed an important divergence in the growth curves after a rapid initial growth (ages <5 yr). Differences in *L_inf_* estimates between colour patterns were as much as 11 cm which represents an increase of ∼31% in spotted individuals. *K* values were higher for plain and female individuals, indicating more rapid convergence on their asymptotic sizes (but do not necessarily grow more rapidly) than spotted individuals and males, respectively. The same conclusions can be drawn from the unconstrained fit of the VBGF, although a complete comparison between the two fits was not possible as the models failed to converge in some cases. The constrained and unconstrained fits of the VBGF to the size-at-age data likely provided an indication of the extremes of the growth pattern for *L. bergylta*, although the true growth pattern most likely lies somewhere between the two fits.

Size structures revealed that spotted individuals were larger than plain individuals, which is in agreement with the differences in the described growth pattern. However, the age distributions completely overlapped, with both plain and spotted individuals present in almost all age classes. The hypothesis of a colour shift with age [56] is thus improbable. The sex-specific size and age distributions support [57] the conclusion of protogyny in *L. bergylta* [13], [20], and the same conclusion of protogyny can be drawn for each colour if they are considered separately.

The observed larger mortality in plain individuals (∼1.5 larger than in spotted individuals) and in females (∼1.6 larger than in males) may be related to size-related predation risk (i.e. reduction of predation with size [58]). Spotted individuals attain larger sizes-at-age, and would therefore be subject to a lower predation risk during their life span, especially in the male phase, since no colour-based differences in mortality were found within the females. However, the mortality pattern of the species seems to be complex, as indicated by the differences in cohort-specific mortality rates in 8 consecutive year-classes (without any clear temporal pattern). These data suggest that shifts in exploitation occur and that these changes may differentially alter sex ratios and colour patterns. In this regard, a more comprehensive population dynamic model that considers sex change and colour patterns should be conducted.

This study revealed striking colour-based demographic variation between plain and spotted morphotypes of the temperate wrasse *Labrus bergylta*. The observed age-based demographic differences are tightly related to the recently reported reproductive traits of the species. For example, Villegas-Ríos et al. [13] observed a difference of 11.2 cm in the size at 50% sex change (36.0 cm for plain individuals, 47.2 cm for spotted individuals) that may be explained by the larger size-at-age of the spotted individuals. In the same study, it was found that the gonadosomatic index (GSI) of the spotted females was significantly lower than the GSI of the plain females. This is consistent with our results from the point of view of the general theory of energy allocation, which states that a trade-off should exist between the energy allocated to growth and reproduction [59]. For a given size, we have demonstrated that plain individuals attained smaller sizes-at-age likely at the cost of their greater reproductive output [13]. However, the basis of the demographic differences between plain and spotted individuals remains unknown. Do plains and spotted morphotypes belong to reproductively isolated populations of the same species? Are the observed differences related to habitat or diet preferences? On one hand, it has been recognized that the combination of assortative mating and disruptive natural selection on a single trait (for example body colour pattern [60], [61], [62]) can initiate speciation in marine environments, even in the absence of geographic barriers (sympatry) and high gene flow [60], [63], [64]. The benthic spawning and nesting behaviour of the species [65], together with likely local larval retention and low adult dispersion [66] would enhance a potential speciation process [67]. However, only a detailed genetic analysis with specific molecular markers would help to elucidate the existence and degree of divergence or disruptive selection between colour patterns and the presence of gene flow between them [60], [68]. On the other hand, differences in diet composition or habitat preference between plain and spotted individuals are currently unexplored. In the Gulf of Maine some cod individuals are resident and feed primarily on benthic fauna rich in carotenoids, which is thought to provide them the red colouration. Other cod individuals are transient, feed on forage fish and display an olive colouration [9]. Red and olive cods are considered two life-history variants as they also display differences in growth, habitat preferences, and body shape. Differences in movement behaviour between colour patterns of *L. bergylta* have never been reported and plain and spotted individuals seem to completely overlap in their depth ranges and habitat preferences (pers. obs.). Therefore it seems unlikely that differences in habitat utilization would explain the colour-based demographic variation. In addition, a comparative analysis of the diet of plain and spotted *L. bergylta* has never been analysed. In conclusion, although the demographic differences (this study) and the reproductive traits [13] are noticeable, the identity of the colour patterns of *Labrus bergylta* remains unresolved and deserves additional research effort. However, the demographic differences between the two morphotypes reported here should be directly applied to the management of this valuable resource in the NE Atlantic, which is solely regulated by minimum landing sizes. We recommend considering plain and spotted morphotypes as two independent management units throughout the distribution range of *L. bergylta*, at least while the status of the species is unresolved.

## Supporting Information

Table S1 Parameters of the unconstrained Von Bertalanffy growth functions with upper and lower 95% confidence intervals and Akaike Information Criteria (AIC) for each model.
(DOCX)

Table S2 Reparametrized von Bertalanffy growth function (rVBGF) parameters estimates with upper and lower 95% confidence intervals.
(DOCX)

Text S1 (DOCX)
