# Supplementary material for: Demographic Variation between Colour Patterns in a Temperate Protogynous Hermaphrodite, the Ballan Wrasse Labrus bergylta
Source: PLoS One. 2013 Aug 23;8(8):e71591. doi: 10.1371/journal.pone.0071591 (PMC3751953; doi:10.1371/journal.pone.0071591)
Supplement: Table S1 — Parameters of the unconstrained Von Bertalanffy growth functions with upper and lower 95% confidence intervals and Akaike Information Criteria (AIC) for each model. (DOCX) [file pone.0071591.s001.docx]

**Table S1.** Parameters of the unconstrained Von Bertalanffy growth functions with upper and lower 95% confidence intervals and Akaike Information Criteria (AIC) for each model

| **Model** | ***L_inf_* - lower** | ***L_inf_*** | ***L_inf_* - upper** | ***K* - lower** | ***K*** | ***K* - upper** | ***T_0_* - lower** | ***T_0_*** | ***T_0_* - upper** | **AIC** |
| --- | --- | --- | --- | --- | --- | --- | --- | --- | --- | --- |
| All | 38.53 | 39.51 | 40.76 | 0.27 | 0.34 | 0.42 | -1.28 | -0.42 | 0.17 | 5195 |
| Females | 35.85 | 36.61 | 37.52 | 0.48 | 0.58 | 0.70 | 0.22 | 0.62 | 0.92 | 3220 |
| Males | na | na | na | na | na | na | na | na | na | na |
| Plain | 35.80 | 36.32 | 36.89 | 0.42 | 0.50 | 0.58 | -0.09 | 0.36 | 0.70 | 3280 |
| Spotted | 50.39 | 53.27 | 57.12 | 0.13 | 0.17 | 0.21 | -2.50 | -1.46 | -0.68 | 1463 |
| Plain females | 33.72 | 34.37 | 35.14 | 0.52 | 0.64 | 0.78 | 0.23 | 0.66 | 0.96 | 1782 |
| Plain males | na | na | na | na | na | na | na | na | na | na |
| Spotted females | 40.22 | 42.04 | 44.54 | 0.30 | 0.41 | 0.53 | -0.44 | 0.28 | 0.77 | 1239 |
| Spotted males | 51.38 | 53.59 | 56.61 | 0.17 | 0.24 | 0.32 | -1.39 | 0.16 | 1.05 | 182 |

na: the model failed to converge
