# Supplementary material for: Demographic Variation between Colour Patterns in a Temperate Protogynous Hermaphrodite, the Ballan Wrasse Labrus bergylta
Source: PLoS One. 2013 Aug 23;8(8):e71591. doi: 10.1371/journal.pone.0071591 (PMC3751953; doi:10.1371/journal.pone.0071591)
Supplement: Table S2 — Reparametrized von Bertalanffy growth function (rVBGF) parameters estimates with upper and lower 95% confidence intervals. (DOCX) [file pone.0071591.s002.docx]

**Table S2** Reparametrized von Bertalanffy growth function (rVBGF) parameters estimates with upper and lower 95% confidence intervals

|  | ***L(2)-*lower** | ***L(2)*** | ***L(2)-*upper** | ***L(5)-*lower** | ***L(5)*** | ***L(5)-*upper** | ***L(8)-*lower** | ***L(8)*** | ***L(8)-*upper** |
| --- | --- | --- | --- | --- | --- | --- | --- | --- | --- |
| All | 20.84 | 22.26 | 23.59 | 32.95 | 33.33 | 33.71 | 36.94 | 37.30 | 37.63 |
| Plain | 18.79 | 20.33 | 21.79 | 32.41 | 32.74 | 33.09 | 35.22 | 35.52 | 35.80 |
| Spotted | 21.60 | 23.44 | 25.04 | 34.69 | 35.23 | 35.80 | 41.65 | 42.36 | 43.03 |
| Plain females | 18.33 | 19.84 | 21.31 | 31.88 | 32.24 | 32.61 | 33.48 | 34.06 | 34.58 |
| Spotted females | 19.05 | 21.15 | 23.23 | 35.27 | 35.86 | 36.47 | 39.36 | 40.21 | 41.14 |
