# Supplementary material for: Demographic Variation between Colour Patterns in a Temperate Protogynous Hermaphrodite, the Ballan Wrasse Labrus bergylta
Source: PLoS One. 2013 Aug 23;8(8):e71591. doi: 10.1371/journal.pone.0071591 (PMC3751953; doi:10.1371/journal.pone.0071591)
Supplement: Text S1 — (DOCX) [file pone.0071591.s003.docx]

**Text S1**

Reparametrized von Bertalanffy growth function method

The reparametrized version of the VBGF equation (rVBGF) describes growth in a similar way as the traditional VBGF and it is based on three parameters, *L(α)*, *L(β)*, *L(γ)* which express the expected average body size at three arbitrary ages (*α*, *β* and *γ*). The biological significance of the parameters allows for a direct comparison of mean size-at-age data between populations. Ages *α*, and *γ* are chosen arbitrarily within the range of ages of the dataset so as to represent the general form of the growth, and *β* is the average age of *α*, and *γ*. The rVBGF equation is as follows:

$$L_{t}=L(\alpha)\frac{\left[ L\left( \gamma\right)-L(\alpha) \right]\left[ 1-r^{\left( 2\left( \frac{t-\alpha}{\gamma-\alpha} \right) \right)} \right]}{1-r^{2}}$$

where

$$r=\frac{L\left( \gamma\right)-L(\beta)}{L\left( \beta\right)-L(\alpha)}$$

and L(t) is the size-at-age, subject to the constraints that *L(α) < L(β) < L(γ)* and *(L(γ) - L(β)) = (L(β) - L(α))*. Estimates of *L(α)*, *L(β)*, *L(γ)* were obtained using the *nlstools* (Baty & Delignette-Muller 2011) and the *FSA* (Ogle 2012) packages. We specifically compared the rVBGF parameters between plains and spotted individuals. Differences between groups were explored based on the overlap of the bootstrapped confidence intervals of the parameters.

Reparametrized von Bertalanffy growth function results

For the rVBGF, we set *α* to age 2 (early fast growth phase) for all groups and *γ* to age 8 (slower growth) for all groups to allow for comparisons. Therefore *β* equalled 5 years. Due to the low number of males of ages 2, we decided to compute the parameters of the rVBGF only for the following groups: all individuals, plain, spotted, plain females and spotted females. There was no differences in *L(2)* in all the five groups analysed, based on bootstrapped 95% confidence intervals (Table S2). We detected significant differences in *L(5)* between plain and spotted individuals, and between plain and spotted females. Those differences were even more noticeable in the case of *L(8)* (Table S2). These results are in agreement with the output of the classic VBGF, which revealed a parallel initial rapid growth in all the groups analysed followed by divergent growth curves between colour patterns and sexes for ages older than 4-5 years.
